# Right Superior Orbitofrontal Gray Matter Volume Reduction Mediates Executive Dysfunction in Schizophrenia Patients With Comorbid Metabolic Syndrome

**DOI:** 10.31083/AP52539

**Published:** 2026-10-16

**Authors:** Hangyu Li, Xinyan Wu, Xinyue Chen, Ying Li, Shiji Peng, Rui Yu, Nian Liu

**Affiliations:** ^1^Department of Radiology, Affiliated Hospital of North Sichuan Medical College, 637000 Nanchong, Sichuan, China; ^2^Nuclear Medicine and Radiation Safety Key Laboratory of Sichuan Province, North Sichuan Medical College, 637000 Nanchong, Sichuan, China

**Keywords:** schizophrenia, metabolic syndrome, gray matter volume, cognitive function, executive function, mediation analysis

## Abstract

**Background:**

Patients with schizophrenia (SCZ) are at a markedly increased risk of developing metabolic syndrome (MetS), which substantially reduces life expectancy and adversely affects treatment adherence. Although cognitive impairment is a core feature of SCZ, the neurobiological mechanisms linking comorbid MetS to cognitive dysfunction remain complex and incompletely understood. Previous studies have primarily focused on the effects of abnormal brain function and white matter abnormalities on cognition, whereas the contribution of gray matter volume (GMV) alterations in the context of comorbid MetS has received comparatively less attention. Accordingly, this study examined GMV alterations in patients with SCZ and comorbid MetS and evaluated whether these alterations mediate the association between MetS and cognitive impairment.

**Methods:**

Sixty clinically stable patients with SCZ receiving risperidone or clozapine were recruited, including 28 meeting the diagnostic criteria for MetS (SCZ-MetS) and 32 without MetS (SCZ-nMetS), together with 31 demographically matched healthy controls (HCs). High-resolution structural magnetic resonance imaging (MRI) scans were analyzed using voxel-based morphometry (VBM) to quantify GMV. Mediation analyses were performed to determine whether GMV alterations mediated the association between MetS and cognitive function.

**Results:**

Compared with the HC group, both the SCZ-nMetS and SCZ-MetS groups exhibited widespread GMV reductions involving the cingulate cortex, left temporal lobe, frontal regions, and insula. Compared with the SCZ-nMetS group, the SCZ-MetS group showed significantly reduced GMV in the right superior orbitofrontal gyrus, as well as in the left amygdala, left insula, and temporal lobe (*p* < 0.05). Mediation analysis indicated that reduced GMV in the right superior orbitofrontal gyrus significantly mediated the association between MetS and deficits in executive function (*p* < 0.05).

**Conclusions:**

MetS-related executive dysfunction may be partly associated with reduced GMV in the right superior orbitofrontal gyrus, suggesting a potential mediating role of this brain region.

## Main Points

1. Metabolic syndrome (MetS) is associated with more extensive gray matter volume (GMV) reductions in schizophrenia patients treated with risperidone or clozapine, particularly in the right superior orbitofrontal gyrus, left insula, and left amygdala.

2. Reduced GMV in the right superior orbitofrontal gyrus mediates the association between MetS and executive dysfunction in schizophrenia patients with comorbid MetS.

3. The right superior orbitofrontal gyrus may serve as a potential structural imaging marker for MetS‑related cognitive impairment in schizophrenia, offering a target for early intervention.

## 1. Introduction

Schizophrenia (SCZ) is a chronic and disabling psychiatric illness marked by positive and negative symptoms and prominent cognitive deficits. Patients with SCZ have a 2.5-fold higher risk of premature death and a 15–20 year reduction in life expectancy compared with the general population [1,2]. Lifelong antipsychotic therapy is necessary. Second-generation antipsychotics (SGAs) remain the mainstay of treatment because they alleviate positive and negative symptoms, improving cognitive functions such as motor processing and executive function [3,4,5], and causing fewer extrapyramidal adverse effects [6,7,8,9]. However, prolonged SGAs exposure, particularly to risperidone and clozapine, substantially increases metabolic syndrome (MetS) risk [10]. MetS prevalence in SCZ is roughly double that of the general population, encompassing central obesity, lipid abnormalities, insulin resistance, hypertension, and type 2 diabetes [10]. These metabolic disturbances further shorten life expectancy and reduce adherence to treatment [11]. Thus, elucidating the neurobiological basis of MetS in the context of long-term antipsychotic therapy is critical for understanding disease progression and improving prognosis in SCZ.

Prior studies have consistently shown that prolonged SGA exposure is associated with weight gain and increased MetS prevalence [12,13]. Emerging evidence also links SGA-related brain changes to metabolic disturbances [14,15]. For example, in patients with early-stage SCZ, reduced gray matter volume (GMV) in the right medial frontal cortex was positively correlated with high-density lipoprotein levels and negatively correlated with body mass index [16]. After 6 weeks of SGAs treatment for SCZ, heightened right putamen reward responses predicted subsequent weight gain [17]. Increased activation in food-reward networks, including the middle and superior frontal gyri, ventromedial and lateral prefrontal cortices, and orbitofrontal cortex, has been linked to weight gain [18]. Collectively, these findings suggest that metabolic abnormalities not only reflect peripheral metabolic dysregulation but may also be closely related to central reward regulation and brain structural remodeling.

Beyond brain structure and function, metabolic abnormalities may further impair cognitive performance. Several studies have reported more severe impairment of executive functioning, verbal memory, and psychomotor speed in patients with SCZ-MetS compared with SCZ-nMetS [19,20], with deficits worsening over time [21]. However, other studies have found no significant cognitive differences [22,23], and some even reported cognitive benefits. This inconsistency may stem from the fact that most studies have focused on behavioral assessments, while the underlying neural mechanisms remain underexplored. Previous studies indicate that structural brain damage and functional abnormalities, particularly GMV reduction, are key neuroimaging markers of cognitive dysfunction [24,25]. Notably, SCZ‑MetS patients exhibit more pronounced reductions in total brain volume and GMV than SCZ‑nMetS patients, especially in the insula and orbitofrontal cortex [26]. Despite these pairwise associations among MetS, GMV, and cognitive function, systematic investigations integrating all three domains are lacking [24,25]. Consequently, the potential pathological cascade from MetS to GMV changes and subsequent cognitive impairment remains poorly understood.

To address this gap, this study applied voxel‑based morphometry (VBM) to measure GMV in SCZ patients receiving risperidone or clozapine, with or without MetS. We tested the mediation hypothesis that GMV mediates the MetS-cognition relationship, such that MetS-associated GMV reduction in key brain regions predicts worse cognitive performance. Testing this hypothesis is essential not only to clarify the neurobiological pathway from metabolic disturbances to cognitive decline in SCZ, but also to identify potential structural targets for early intervention aimed at mitigating MetS‑related brain and cognitive sequelae.

## 2. Material and Methods

### 2.1 Participants

Individuals identified with SCZ were recruited consecutively from the inpatient departments of Bashu Psychiatric Hospital and Ruikang Psychiatric Specialty Hospital (Wusheng County, Guang’an, Sichuan Province, China), where they were receiving ongoing clinical care. The validation of SCZ diagnoses was conducted independently by a pair of board-certified psychiatrists using a structured clinical interview that followed the criteria outlined in the diagnostic and statistical manual of mental disorders-5 (DSM-5).

Individuals without mental healthy controls (HC) from the same community were enlisted via public announcements. To confirm that these HC participants had no psychiatric conditions, qualified clinicians conducted the non-patient version of the structured clinical interview for DSM-IV axis I disorders (SCID-NP). Additionally, all HCs reported no history of psychiatric disorders in their first-degree relatives. To ensure metabolic comparability, HCs with MetS were excluded using metabolic indicators obtained from routine physical examinations performed within the previous three months.

### 2.2 Inclusion and Exclusion Criteria

Inclusion criteria for the SCZ groups were: (1) right-handedness; (2) Han Chinese ethnicity; (3) current treatment with either risperidone or clozapine; (4) illness duration of at least two years as corroborated by family informants, hospital records, and the Nottingham Onset Schedule; (5) age ≥18 years.

Exclusion criteria for both the SCZ and HC groups comprised: (1) alcohol or substance misuse; (2) severe systemic illnesses such as malignancy or cardiovascular disease; (3) history of head injury or other structural brain lesions; (4) contraindications to magnetic resonance imaging (MRI). An experienced neuroradiologist inspected all MRI datasets to rule out overt structural brain abnormalities.

### 2.3 Diagnostic Criteria for MetS

MetS was diagnosed based on the national cholesterol education program adult treatment panel III (NCEP ATP III) criteria with ethnicity‑specific adaptations for Chinese individuals [27]. Participants were classified as having MetS if they fulfilled at least three of the following criteria: (1) fasting serum triglycerides (TG) ≥150 mg/dL (1.7 mmol/L); (2) waist circumference ≥90 cm for men or ≥80 cm for women; (3) fasting blood glucose (FBG) ≥100 mg/dL (5.6 mmol/L) or a clinical diagnosis of type 2 diabetes; (4) systolic blood pressure (SBP) ≥130 mmHg and/or diastolic blood pressure (DBP) ≥85 mmHg, or antihypertensive treatment; (5) low high-density lipoprotein cholesterol (HDL-C) <40 mg/dL (1.03 mmol/L) in men or <50 mg/dL (1.29 mmol/L) in women, or use of lipid-lowering medication.

### 2.4 Sample Size Calculation and Demographic Characteristics

A sample size calculation was performed using G*Power 3.1 software (Heinrich Heine University Düsseldorf, Düsseldorf, Germany). With an expected effect size of 0.4 and a significance level of 0.05, a minimum total of 64 participants would be required to achieve 80% statistical power. The final study included 31 healthy controls and 60 patients diagnosed with SCZ. A post hoc power analysis revealed that the achieved statistical power for the current sample size was 0.9284, confirming that the study was adequately powered. Participants were divided into three groups: (1) SCZ with MetS (SCZ-MetS; n = 28, 11 men and 17 women); (2) SCZ without MetS (SCZ-nMetS; n = 32, 22 men and 10 women); and (3) HC (n = 31, 15 men and 16 women). The three groups showed no significant differences regarding age, distribution of sex, or level of education. In the SCZ-MetS group, 8 patients were on risperidone monotherapy, 4 on clozapine monotherapy, and 16 on a combination of both, whereas in the SCZ-nMetS group, 12 were on risperidone monotherapy, 2 on clozapine monotherapy, and 18 on a combination of both. Antipsychotic doses were converted to chlorpromazine‑equivalent values for standardization [28]. MetS status was ascertained using admission records and laboratory results obtained at the time of hospitalization.

### 2.5 Clinical Data Acquisition

Consistent with the MetS criteria outlined above, all participants received a comprehensive metabolic evaluation, including triglycerides (TG), body mass index (BMI), fasting blood glucose (FBG), high‑density lipoprotein (HDL), low‑density lipoprotein (LDL), blood pressure (BP), total cholesterol (TC), and a documented history of MetS. BP was measured in a seated position using an automated digital sphygmomanometer. BMI was calculated as weight in kilograms divided by height in meters squared [3]. Venous blood samples were obtained after an 8‑hour fasting period for quantification of HDL, LDL, TC, and TG. Waist circumference was measured at the midpoint found between the lower edge of the rib cage and the upper part of the hip bone. All metabolic and anthropometric data were collected during the same clinical session to minimize variability.

Cognitive abilities of all participants were evaluated using the ‘brief assessment of cognition in schizophrenia’ (BACS) [29], which comprises six cognitive domains represented by seven tests: verbal memory (List Learning), motor speed (Token Motor), executive function (Tower of London), attention/processing speed (Symbol Coding), working memory (Digit Sequencing), and verbal fluency (Category Fluency and Letter Fluency) [29]. The ‘Rating Scale for Extrapyramidal Side Effects’ (RSESE) [30] was utilized to evaluate extrapyramidal symptoms. Additionally, adverse drug reactions were examined using the ‘treatment emergent symptom scale’ (TESS) [31], which comprises 34 items covering specific symptoms and general queries rated on a four‑point scale, with higher scores reflecting more severe treatment‑emergent effects. Importantly, all of the above clinical and cognitive scales are well validated for Chinese populations [22].

### 2.6 Image Data Acquisition

Neuroimaging was conducted using a 3.0-Tesla MRI system (Discovery MR750, GE Healthcare, Milwaukee, WI, USA) that had a 32-channel phased-array head coil. To minimize scanner noise, participants were provided with earplugs, and foam padding was utilized to limit head motion. Every participant underwent scanning according to the same imaging protocol. A high-resolution 3D T1-weighted sequence was acquired as follows: field of view [FOV] = 256 × 256 mm^2^; flip angle = 12°; repetition time [TR] = 8.5 ms; isotropic voxel size = 1 × 1 × 1 mm^3^; 188 contiguous axial slices; echo time [TE] = 2.0 ms; and matrix size 256 × 256.

### 2.7 Data Processing and Voxel-Based Morphometry

Analyses utilizing VBM were performed with the computational anatomy toolbox (CAT12, University of Jena, Jena, Germany) as incorporated in SPM12 (Wellcome Department of Imaging Neuroscience, London, UK) [32,33]. High-resolution T1-weighted images were adjusted for bias-field inhomogeneity, categorized into gray matter (GM), white matter (WM), and cerebrospinal fluid (CSF), and then spatially standardized to a reference space applying the DARTEL algorithm. Partial volume effects were accounted for following established procedures. To minimize misclassification artifacts at GM/WM boundaries, an absolute gray matter threshold of 0.1 was applied [32]. Following preprocessing, visual inspection was conducted for all GM images to ensure quality control, and images with marked artifacts were excluded. Following that, GM maps were enhanced through the use of an isotropic Gaussian kernel, which featured a full-width at half-maximum (FWHM) measurement of 8 mm.

### 2.8 Statistical Analysis

Statistical analyses were conducted with the use of R 4.2.1 (R Foundation for Statistical Computing, Vienna, Austria), and a significance level of *p* < 0.05 was established for two-tailed tests. The Shapiro-Wilk test was utilized to evaluate the normality of both demographic and clinical variables. For continuous variables that followed a normal distribution, either independent-samples *t*-tests or one-way ANOVAs were applied, while categorical variables were analyzed using the chi-square test. In the case of skewed variables, the Mann-Whitney U test or the Kruskal-Wallis test was used. Data that are normally distributed are presented as means ± standard deviation, while skewed data are expressed as median values along with interquartile ranges.

In the VBM analyses, one-way ANCOVAs were performed at the voxel level among the three groups (SCZ-MetS, SCZ-nMetS and HC), incorporating age, sex, years of education, and total intracranial volume as covariates. To control for false positives in whole-brain voxel-wise inference (involving hundreds of thousands of tests), we used a conservative two-step correction: an initial voxel-wise threshold of *p* < 0.001 (uncorrected), followed by cluster-level family-wise error (FWE) correction (*p* < 0.05). Only clusters exceeding 36 voxels were retained for subsequent analyses [32,33]. Significant clusters were subjected to post hoc pairwise comparisons.

Subsequently, partial correlations were conducted between GMV in regions with significant intergroup differences (SCZ‑MetS vs. SCZ‑nMetS) and metabolic/cognitive indicators, controlling for age, sex, education years, total intracranial volume, and medication dose. FDR correction was applied for multiple comparisons.

A mediation model was constructed to investigate the mediating role of GMV alterations between MetS status and cognitive dysfunction. In this model, MetS status was specified as the independent variable (X), GMV in brain regions showing significant differences between patient subgroups as the mediator (M), and cognitive function scores as the dependent variable (Y). Age, sex, years of education, total intracranial volume, and medication dosage were included as covariates. A bias-corrected bootstrap mediation analysis (5000 iterations) was conducted using the PROCESS macro version 4.1 (Andrew F. Hayes, The Ohio State University, Columbus, OH, USA) to test the indirect effect. The point estimate of the indirect effect was considered statistically significant if its 95% bootstrap confidence interval did not include zero.

## 3. Results

### 3.1 Participant Characteristics

No significant differences were observed among the SCZ-MetS, SCZ-nMetS, and HC groups in years of education, sex distribution, age, LDL, TC, or HDL levels. Within the SCZ patients, the MetS and non-MetS subgroups did not differ significantly in illness duration, daily antipsychotic dosage converted to chlorpromazine equivalents, BACS composite scores, or scores on the RSESE and TESS. Compared with HC, both SCZ-MetS and SCZ-nMetS groups demonstrated markedly lower cognitive performance on all BACS measures, while the SCZ-MetS group additionally showed significantly higher levels of blood glucose, TG, waist circumference, and hip circumference. The SCZ-MetS group displayed higher waist and hip circumferences, SBP, DBP, FBG, and TG levels than the SCZ-nMetS group, whereas no significant differences were found between the two patient groups in any of the BACS cognitive scores or other demographic/clinical variables (Table 1).

**Table 1.** **Participant demographics and clinical characteristics**.

Characteristic	SCZ-MetS	SCZ-nMetS	HC	*p*-value	Post hoc analysis		
	(N = 28)	(N = 32)	(N = 31)		G1-2	G1-3	G2-3
Age (years)	41.4 ± 10.2*&*	35.7 ± 9.5	39.9 ± 8.1	0.052^a^	/	/	/
Sex (M/F)	11, 17	22, 10	15, 16	0.062^b^	/	/	/
Education years (years)	7.5 (6, 9)*#*	8.5 (6, 9)	9 (7, 11)	0.065^a^	/	/	/
Illness duration (years)	8.5 (5, 11)	12.2 ± 6.5	/	0.064^e^	/	/	/
Medication dosage (chlorpromazine equivalent)	200 (139.5, 284.2)	237.5 (200, 374)	/	0.192^e^	/	/	/
List Learning	18.0 ± 11.8	19.6 ± 9.6	37.0 ± 11.7	<0.001^a^	1.000	<0.001	<0.001
Symbol Coding	18.2 ± 13.4	19.8 ± 11.7	44 ± 19.6	<0.001^a^	1.000	<0.001	<0.001
Verbal Fluency	16.9 ± 10.9	17.4 ± 7.0	25.4 ± 9.2	<0.001^a^	1.000	<0.001	<0.001
Token Motor Task	57.8 ± 20.2	55.0 ± 22.0	82 (74, 88)	<0.001^c^	1.000	<0.001	<0.001
Tower of London Test	9.5 ± 7.1	11 (3, 15.8)	19 (15, 21)	<0.001^c^	1.000	<0.001	<0.001
Digit Sequencing Task	16 (5, 16)	16 (8, 20)	24 (23, 27)	<0.001^c^	1.000	<0.001	<0.001
BACS Global score	133.18 ± 54.5	136.1 ± 50.4	227.5 ± 43	<0.001^a^	1.000	<0.001	<0.001
Low density lipoprotein (mmol/L)	2.9 ± 0.7	2.9 ± 0.1	2.9 ± 0.8	0.993^a^	/	/	/
Total cholesterol (mmol/L)	4.4 ± 0.9	4.1 ± 0.9	4.6 (3.6, 5.0)	0.315^c^	/	/	/
Fasting blood glucose levels (mmol/L)	5.9 (5.2, 6.7)	4.5 (4.2, 5.3)	4.8 ± 0.6	0.001^c^	0.005	0.004	1.000
High-density lipoprotein (mmol/L)	1.3 ± 0.6	1.4 ± 0.6	1.2 (0.9, 1.4)	0.138^c^	/	/	/
Systolic blood pressure (mmHg)	126.9 ± 17.1	110.8 ± 14.1	/	<0.001^d^	/	/	/
Body mass index (kg/m^2^)	24.9 ± 3.4	22.1 ± 3.4	23.8 ± 3.2	0.006^a^	0.005	0.600	0.100
Diastolic blood pressure (mmHg)	82 ± 11.0	74.7 ± 7.7	/	0.008^d^	/	/	/
Triglycerides (mmol/L)	2.0 (1.7, 2.5)	1.6 (1.3, 2.0)	1.6 (1.4, 1.7)	<0.001^c^	0.002	0.002	1.000
Waist circumference (cm)	91.5 ± 6.3	81.1 ± 9.2	82.1 ± 5.7	<0.001^a^	<0.001	<0.001	1.000
Hip circumference (cm)	96.4 ± 5.5	91.4 ± 6.0	90.6 ± 7.8	0.002^a^	0.011	0.003	1.000
TESS	6.2 ± 5.7	6 (2, 9)	/	0.623^e^	/	/	/
RSESE	0 (0, 1.8)	0 (0, 3)	/	0.447^e^	/	/	/

**Note 1:** # Values are shown as median (first quartile, third quartile); & Values are expressed as mean ± SD. Post-hoc *t*-test *p*-value was corrected using Bonferroni; ^a^: one-way ANOVA; ^b^: chi-square analysis; ^c^: Kruskal-Wallis H-test; ^d^: two-sample *t*-test; ^e^: Mann-Whitney U test; G1-2: SCZ-MetS vs SCZ-nMetS groups; G1-3: SCZ-MetS vs HC groups; G2-3: SCZ-nMetS vs HC groups. SCZ, schizophrenia; MetS, metabolic syndrome; HC, healthy controls; BACS, brief assessment of cognition in schizophrenia; TESS, treatment emergent symptom scale; RSESE, rating scale for extrapyramidal side effects.

In addition, due to the different distribution patterns of disease duration between patient subgroups, a post-hoc sensitivity analysis was conducted to control for its potential impact. The results revealed no significant association between disease duration and our findings (**Supplementary Fig. 1**).

### 3.2 GMV Comparisons Among Groups

#### 3.2.1 Regional GMV Alterations Across the Three Groups

Regions of the brain showing notable differences in GMV across the three groups include the bilateral medial cingulate cortex, left anterior and paracingulate gyri, left middle frontal gyrus, right inferior frontal gyrus (pars opercularis), right dorsolateral superior frontal gyrus, bilateral insula, right superior and middle orbitofrontal gyri, left amygdala, left middle temporal gyrus, and right superior and inferior temporal gyri (Table 2).

**Table 2.** **Gray matter differences among the three groups and post hoc analysis**.

Brain region		Cluster size	F value	MNI coordinate	Post hoc analysis		
				x y z	G1-2	G1-3	G2-3
*L*	Middle temporal gyrus	219	11.94	(–50, –60, –2)	<0.001^a^	<0.001^a^	0.479^a^
	Insula	96	16.98	(–39, 15, 9)	0.040^a^	<0.001^a^	0.011^a^
	Amygdala	184	20.03	(–29, 5, –20)	0.017^a^	<0.001^a^	<0.001^a^
	Middle frontal gyrus	157	9.73	(–27, 42, 28)	0.152^a^	<0.001^a^	0.011^a^
	Median cingulate and paracingulate gyri	175	13.42	(–10, 14, 40)	0.114^a^	<0.001^a^	0.025^a^
	Anterior cingulate and paracingulate gyri	60	17.85	(–10, 50, –2)	0.060^a^	<0.001^a^	0.011^a^
*R*	Middle orbitofrontal gyrus	223	12.88	(11, 42, –8)	<0.001^a^	<0.001^a^	0.185^a^
	Inferior temporal gyrus	130	12.99	(51, –53, –12)	0.024^a^	<0.001^a^	0.059^a^
	Superior frontal gyrus, dorsolateral	273	11.94	(51, –41, 18)	0.717^a^	<0.001^a^	<0.001^a^
	Superior orbitofrontal gyrus	189	10.93	(11, 57, –14)	0.023^a^	<0.001^a^	0.060^a^
	Median cingulate and paracingulate gyri	108	10.30	(10, 16, 40)	0.025^a^	<0.001^a^	0.074^a^
	Superior temporal gyrus	82	9.60	(24, 8, 54)	<0.001^a^	<0.001^a^	0.185^a^
	Inferior frontal gyrus, opercular part	43	10.41	(38, 10, 27)	0.345^a^	0.017^a^	<0.001^a^
	Insula	85	9.21	(40, –16, 10)	0.210^a^	<0.001^a^	0.022^a^

**Note 2: **R/L: right/left hemisphere; ^a^: FDR-corrected; G1-2: SCZ-MetS vs SCZ-nMetS groups; G1-3: SCZ-MetS vs HC groups; G2-3: SCZ-nMetS vs HC groups. MNI, montreal neurological institute (stereotaxic space).

#### 3.2.2 GMV Differences Between SCZ Subgroups and HCs

Compared with the HC group, both SCZ subgroups exhibited significant GMV reductions in the left anterior cingulate cortex, left medial cingulate cortex, left amygdala, left middle frontal gyrus, right opercular part of the inferior frontal gyrus, right superior frontal gyrus, and bilateral insulae. Additionally, the SCZ-MetS group showed further GMV reductions in the left middle temporal gyrus, right medial cingulate cortex, right orbitofrontal and medial orbitofrontal cortices, as well as the right superior and inferior temporal gyri (Table 2, Figs. 1,2).

**Fig. 1.**
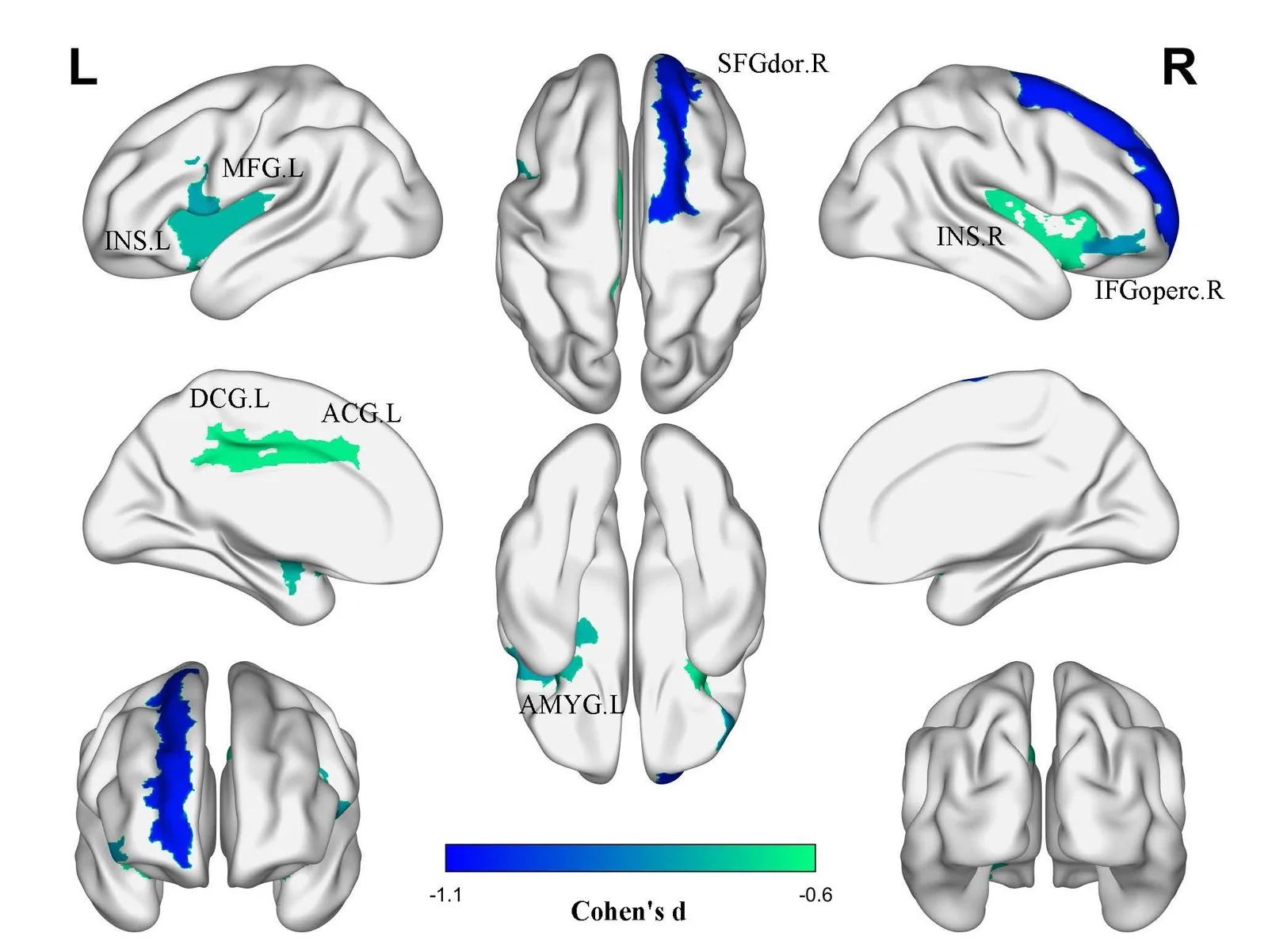
**Gray matter volume alterations between SCZ-nMetS and HC groups**. Note 3: R/L, right/left hemisphere; MFG, middle frontal gyrus; DCG, median cingulate and paracingulate gyri; ACG, anterior cingulate and paracingulate gyri; SFGdor, superior frontal gyrus, dorsolateral; IFGoperc, inferior frontal gyrus, opercular part; AMYGL, amygdala; INS, insula.

**Fig. 2.**
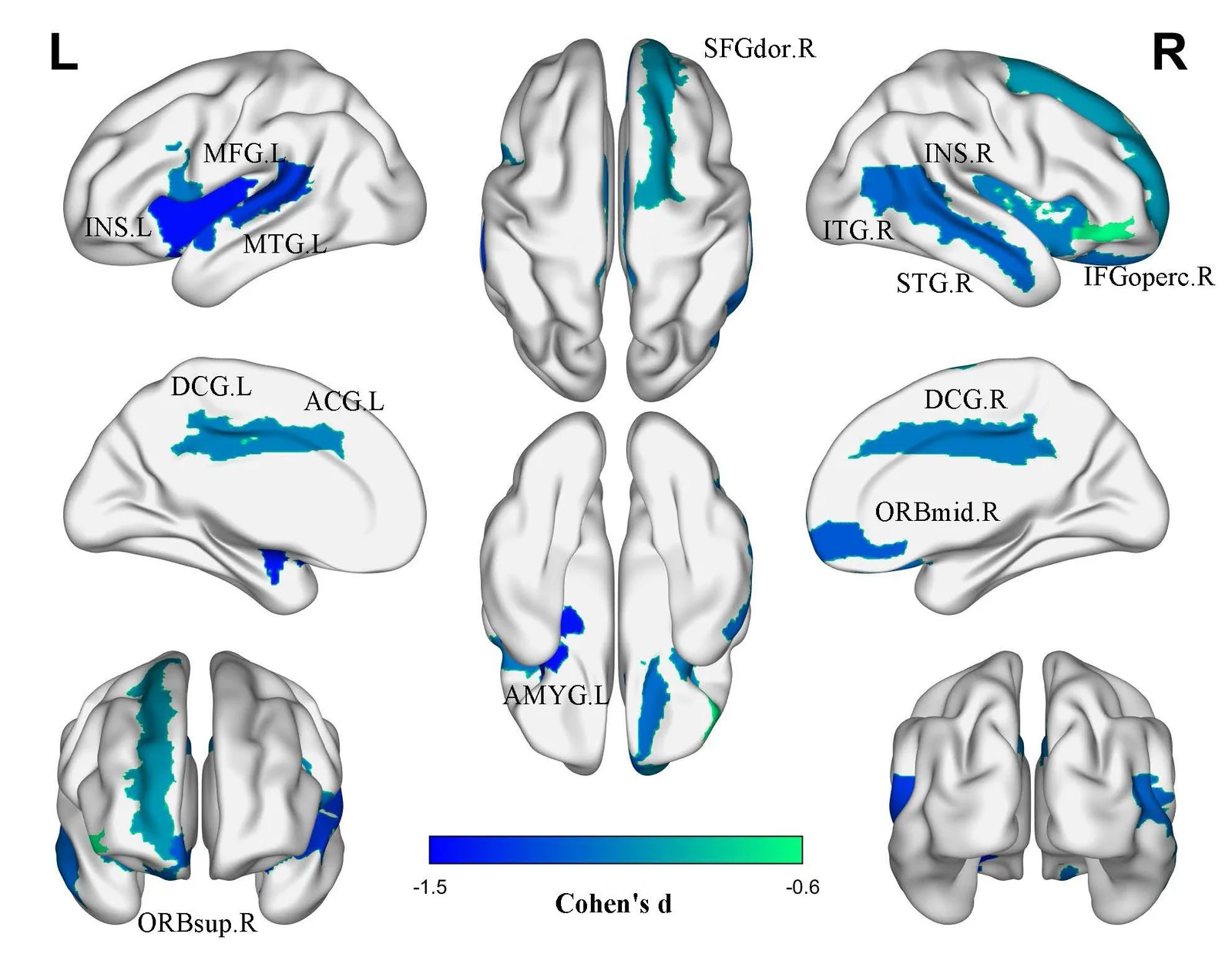
**Gray matter volume differences between SCZ-MetS and HC groups**. Note 4: R/L, right/left hemisphere; STG, superior temporal gyrus; ORBsup, superior orbitofrontal gyrus; ORBmid, middle frontal gyrus, orbital part; MTG, middle temporal gyrus; ITG, inferior temporal gyrus.

#### 3.2.3 GMV Alterations Between SCZ-MetS and SCZ-nMetS Groups

Compared with the SCZ-nMetS group, the SCZ-MetS group showed significantly lower GMV in the left insula, left middle temporal gyrus, left amygdala, the right superior and middle orbitofrontal gyri, the right medial cingulate cortex, and the right superior and inferior temporal gyri (Table 2, Fig. 3). In addition, given the marginal significance of age and sex, which may introduce confounding effects, we performed interaction analyses using a general linear model with GMV and cognitive function scores as dependent variables (Group × Age and Group × Sex). FDR correction was applied. The results showed that neither age nor sex had significant interaction effects on the findings (**Supplementary Table 1**).

**Fig. 3.**
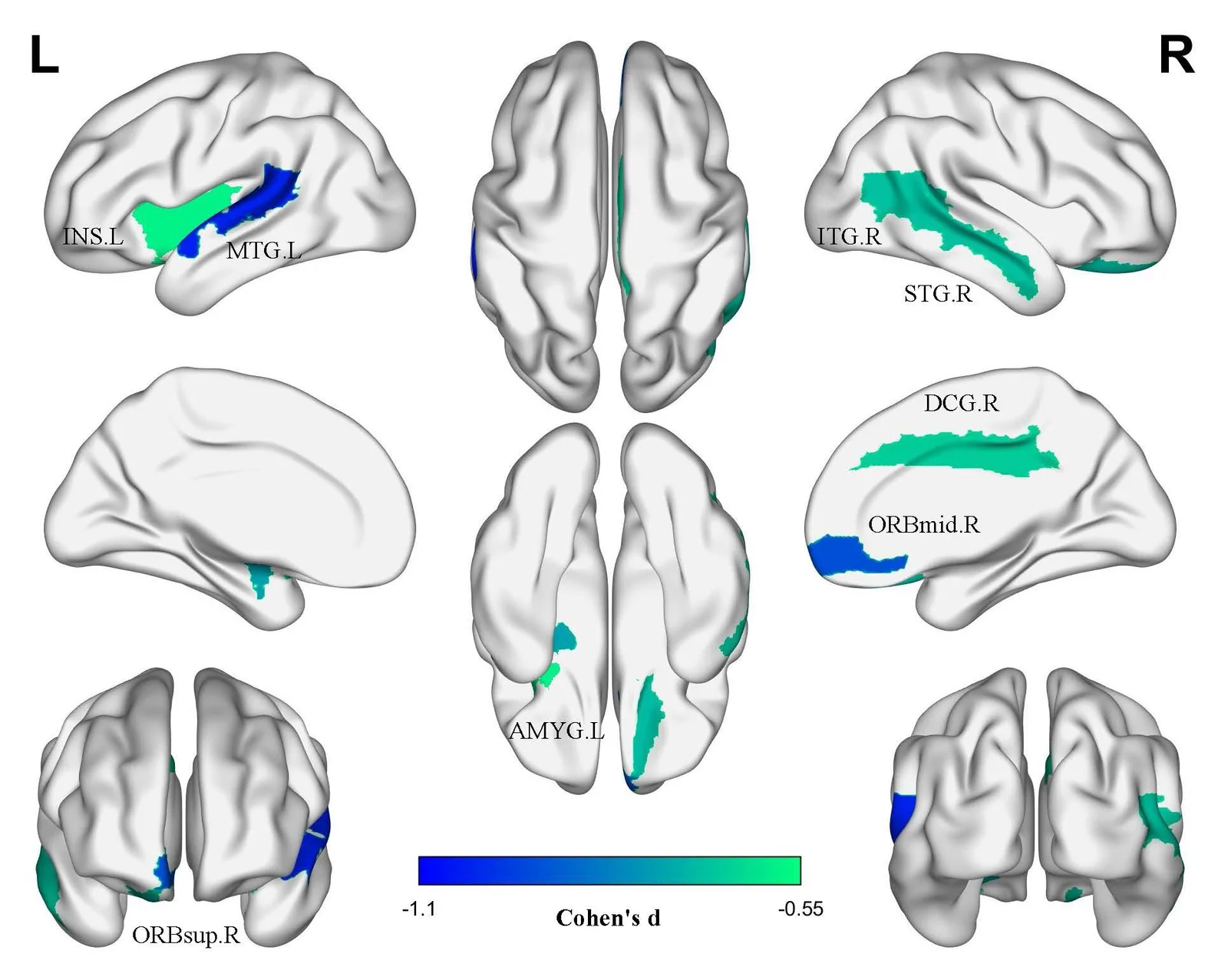
**Gray matter volume differences between SCZ-MetS and SCZ-nMetS groups**.

### 3.3 Correlations Between GMV, MetS Indices, and Cognition

Partial correlation analysis revealed a significant positive correlation between the GMV of the right superior orbitofrontal gyrus and executive function scores (*p *= 0.015, FDR-corrected) (Fig. 4).

**Fig. 4.**
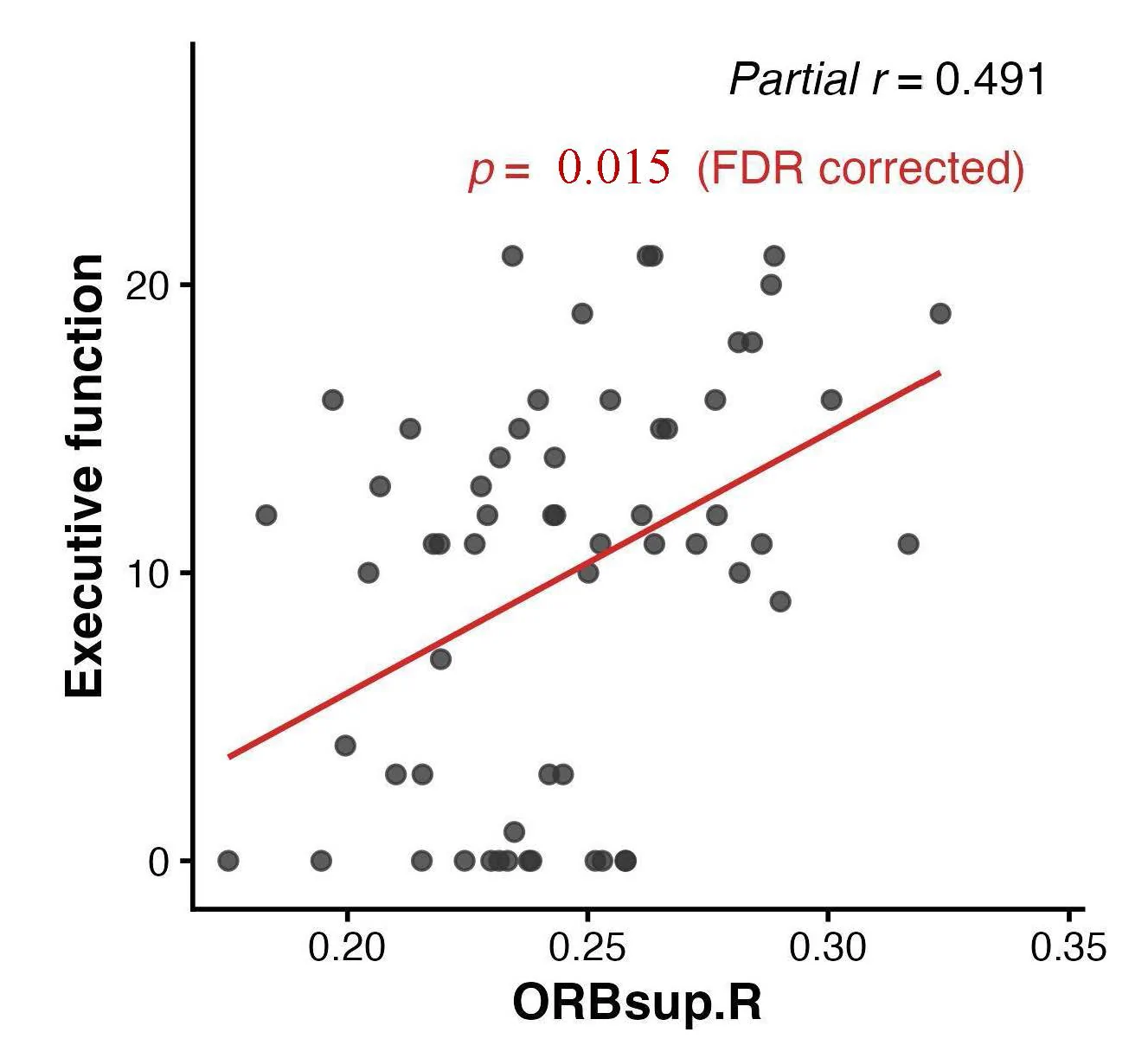
**Associations between Gray matter volume in significantly altered brain regions and clinical indices**.

### 3.4 Mediation Analysis

Only GMV in the right superior orbitofrontal gyrus was significantly positively correlated with executive function scores (FDR corrected). Accordingly, we focused on the mediating role of GMV in this region between MetS and executive function. Mediation modeling in SCZ patients demonstrated a significant negative association between MetS status and GMV in the right superior orbitofrontal gyrus (*p* = 0.009, t = −2.70, β = −0.024, 95% CI = −0.042 to −0.006, SE = 0.009). Moreover, GMV in this region exhibited positive associations with executive function scores (β = 109.460, SE = 21.856, 95% CI = 65.623 to 153.298, t = 5.01, *p* < 0.001) (Fig. 5). Notably, right superior orbitofrontal GMV reduction had a significant indirect negative effect linking MetS to executive dysfunction (indirect effect = −2.627, 95% CI = −4.999 to −0.807, *p* = 0.013, FDR corrected), while the total effect of MetS on executive function was not significant. This pattern indicates an inconsistent mediation (suppression) effect, implying that GMV loss in the right superior orbitofrontal gyrus serves as a key intermediary between metabolic burden and executive dysfunction in SCZ-MetS patients.

**Fig. 5.**
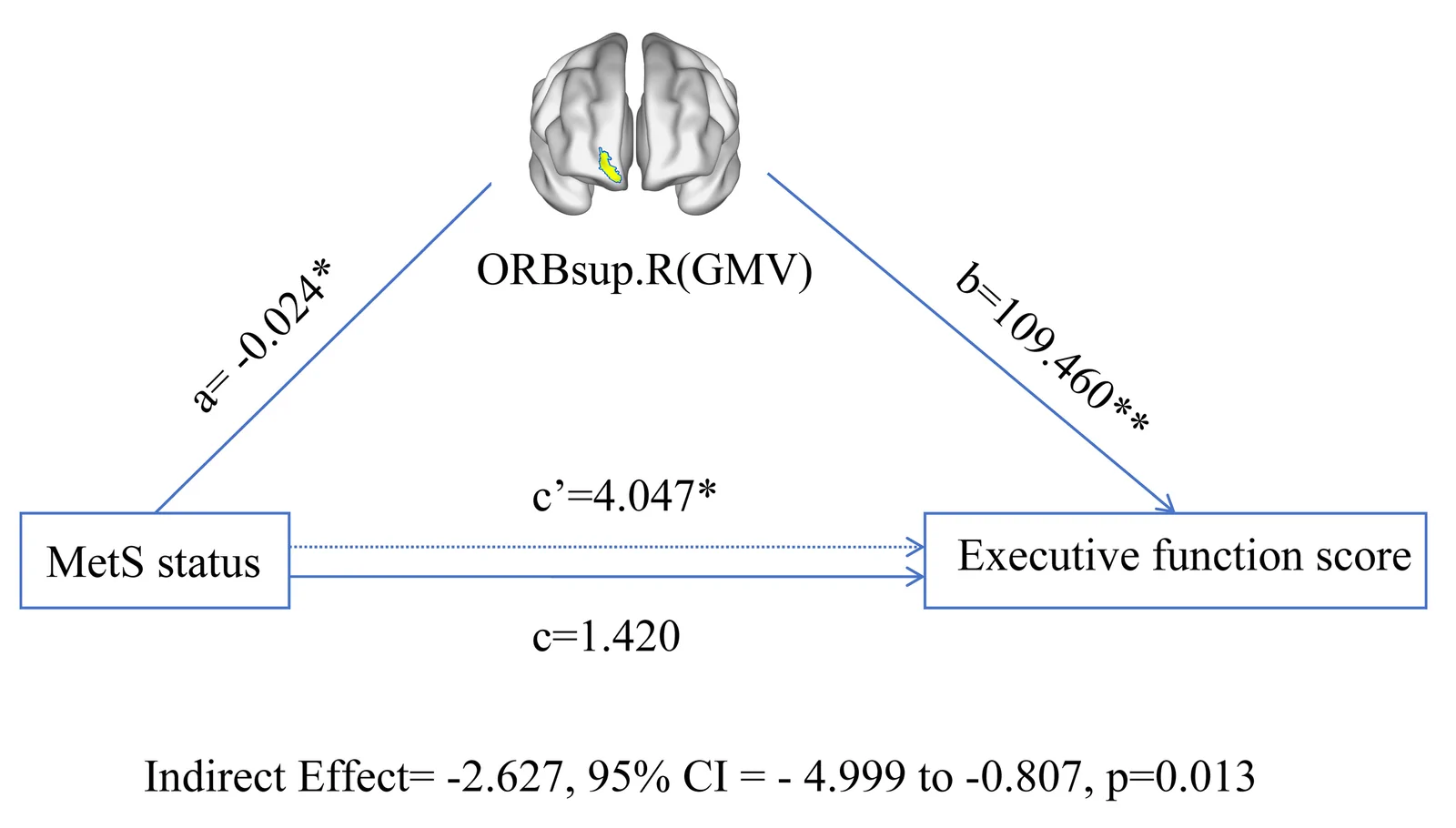
**Mediation analysis**. Note 5: MetS, metabolic syndrome; *: *p *< 0.05; **: *p *< 0.001; ORBsup.R, right superior orbitofrontal gyrus; a: path from the independent variable to the mediator; b: path from the mediator to the dependent variable (controlling for the independent variable); c: total effect of the independent variable on the dependent variable; c’: direct effect of the independent variable on the dependent variable (controlling for the mediator).

## 4. Discussion

This voxel-based morphometry study investigated GMV alterations in SCZ patients with comorbid MetS and revealed that reduced GMV in the right superior orbitofrontal gyrus mediates the relationship between MetS and executive dysfunction. These findings not only provide new neuroimaging evidence for structural brain changes in SCZ-MetS patients but also preliminarily reveal the neuroimaging associations among MetS, GMV, and cognitive function.

### 4.1 MetS Exacerbates GMV Loss in SCZ

Compared with the HC group, both SCZ groups displayed significant GMV reductions in the cingulate, temporal, frontal, and insular cortices. In contrast, the SCZ-MetS group exhibited further reductions in GMV within the left middle temporal gyrus, right medial cingulate cortex, right orbitofrontal and medial orbitofrontal cortices, as well as the right superior and inferior temporal gyri compared to the SCZ-nMetS groups. These observed alterations corroborate prior findings on widespread GM atrophy in SCZ, with pronounced reductions localized to the prefrontal cortex, superior temporal gyrus, insular cortex, and cingulate gyrus [34,35,36]. Furthermore, temporal lobe GMV deficits have been documented in overweight or obese individuals regardless of psychiatric status [37,38], and MetS has been linked to smaller GMV in the broader temporal lobe regions, right superior temporal gyrus, and right insula [39,40]. In summary, our findings confirm core structural abnormalities in the frontal and temporal lobes in SCZ. Moreover, in patients treated with risperidone or clozapine, MetS is associated with more widespread and severe loss of GMV.

### 4.2 Characteristic Neuroanatomical Alterations in SCZ-MetS Patients and Their Associations With Metabolic Dysregulation

Compared with SCZ-nMetS group, individuals in the SCZ-MetS subgroup exhibited pronounced GMV reductions in the right superior orbitofrontal gyrus (a key sector of the orbitofrontal cortex, OFC) and in the left amygdala and insula. Our prior work likewise reported diminished regional homogeneity within the right inferior OFC in SCZ-MetS patients relative to SCZ-nMetS patients [22]. While this study explored the right superior orbitofrontal gyrus, both regions belong to the OFC, suggesting that the OFC may play an important role in the pathophysiology of SCZ-MetS patients. Previous studies have also demonstrated OFC volume loss in SCZ-MetS patients, which is consistent with our findings [26,41]. The OFC integrates sensory, limbic, and prefrontal signals to guide food-related decision-making and is believed to be closely associated with the suppression of maladaptive behaviors such as overeating and addictive tendencies [26,41]. Research involving functional imaging suggests that individuals who are overweight or obese exhibit decreased activity in the OFC and demonstrate increased responsiveness to food-related stimuli [42]. Additionally, previous studies have associated a smaller GMV in the OFC with obesity [43]. OFC hyper-reactivity to food stimuli has also been reported in obesity [42], a major precursor of MetS [44]. In summary, OFC structural and functional variations are statistically associated with both reduced self-control and increased susceptibility to MetS.

The insula and amygdala are central components of the limbic system that integrate interoceptive information such as hunger, satiety, and autonomic feedback, while also orchestrating emotional and stress responses. Prior studies have documented reduced insular GMV in SCZ with obesity [45] and in individuals with MetS [37]. Chronic hyperglycemia and dyslipidemia are hallmarks of MetS and have been theorized by prior research to promote neuroinflammation and oxidative stress, which might correlate with reduced insular and amygdalar GMV. Such variations may further contribute to altered appetite regulation, autonomic imbalance, and heightened stress reactivity. These alterations potentially align with hedonic eating and persistent sympathetic overactivity, which may be associated with the worsening of insulin resistance, obesity, and hypertension [46]. Conversely, progressive GMV loss may further correlate with impaired interoceptive awareness and emotional control, may co-occur with high-calorie intake and dysregulated stress hormone secretion, and may be associated with the worsening of metabolic dysfunction.

Importantly, it must be explicitly acknowledged that the current study utilizes a cross-sectional design. Therefore, while we hypothesize a potential reciprocal interplay between GMV deficits and metabolic deterioration based on the existing literature, our data cannot establish a definitive temporal order or causal pathways. The observed relationships should be strictly interpreted as statistical correlations. Future longitudinal studies are essential to determine whether these neuroanatomical alterations are the consequence of metabolic dysregulation, a contributing factor, or both.

### 4.3 The Mediating Role of Right Superior Orbitofrontal GMV Reduction Between MetS and Executive Dysfunction

Mediation analysis showed a significant negative association between MetS status and right superior orbitofrontal GMV. Reduced GMV in this region mediated a significant indirect negative effect between MetS and executive dysfunction (indirect effect = −2.627), whereas the total effect of MetS on executive function was non-significant. This inconsistent mediation pattern [26,47] suggests that the MetS-cognition link involves opposing pathways: a negative indirect route via OFC structural loss [48] and a potential positive pathway.

Consistent with the total effect of the mediation model, the lack of cognitive differences between patient groups aligns with some previous reports [22,23]. However, other studies have reported [49,50] worse cognitive performance in SCZ-MetS patients compared with SCZ-nMetS patients [51]. Several factors may be associated with these inconsistencies. First, unlike previous studies [49,50], all patients in the present study were treated with risperidone or clozapine, both of which are known to improve cognitive function [3,4,5], which may have attenuated the cognitive differences between groups. Second, previous studies [52,53] have shown that distinct metabolic components differentially affect cognitive function. Together, these possibilities suggest the existence of a potential positive pathway that may improve cognitive function.

In summary, the present study identified a statistical association between MetS and GMV reductions in specific brain regions, particularly the right superior orbitofrontal gyrus. Furthermore, GMV reduction in this region was found to mediate the relationship between MetS and executive dysfunction. Accordingly, we cautiously speculate that, in antipsychotic-treated SCZ patients, comorbid MetS may reduce GMV in key brain regions, partially mediating executive dysfunction. However, this speculation requires validation in future longitudinal studies.

### 4.4 Limitations

Several study limitations warrant consideration. First, the modest sample size underscores the need for replication in larger, independent cohorts. Even so, exploratory analyses can nonetheless reveal meaningful trends regarding MetS-related brain changes. Second, no systematic evaluation of the contributions of individual MetS components to structural and cognitive outcomes was conducted, so future research should examine both component-level and phenotype-level effects. Third, all patients were receiving risperidone or clozapine, limiting generalizability to other antipsychotic treatments. Fourth, the cross-sectional design precludes causal inference regarding the directionality between MetS and GMV loss, highlighting the need for longitudinal follow-up. Finally, age and sex showed marginal significance in this study. Although these potential confounders have been statistically adjusted for, our findings should be validated in larger independent samples.

## 5. Conclusions

In patients with SCZ receiving risperidone or clozapine, comorbid MetS is associated with reduced GMV in the right superior orbitofrontal gyrus, left insula, and amygdala. Moreover, MetS-related executive dysfunction may be partially attributable to GMV reduction in the right superior orbitofrontal gyrus, suggesting a potential mediating role. Future longitudinal studies, multimodal imaging investigations (e.g., resting-state functional magnetic resonance imaging, rs-fMRI; diffusion tensor imaging, DTI; positron emission tomography, PET), and randomized controlled trials combining neuromodulation with metabolic interventions may help to further explore causality and preliminarily assess whether the right superior orbitofrontal gyrus could serve as a possible early imaging marker.

## Data Availability

The data that support the findings of this study are available from the corresponding author upon reasonable request.
