# Supplementary material for: Right Superior Orbitofrontal Gray Matter Volume Reduction Mediates Executive Dysfunction in Schizophrenia Patients With Comorbid Metabolic Syndrome
Source: Alpha Psychiatry. 2026 Oct 16;27(5):52539. doi: 10.31083/AP52539 (PMC13731999; doi:10.31083/AP52539)
Supplement: Supplementary file 1 [file 2757-8038-27-5-52539-s1.zip › Supplementary Material.pdf]

Supplementary Figure 1 Sensitivity Analysis

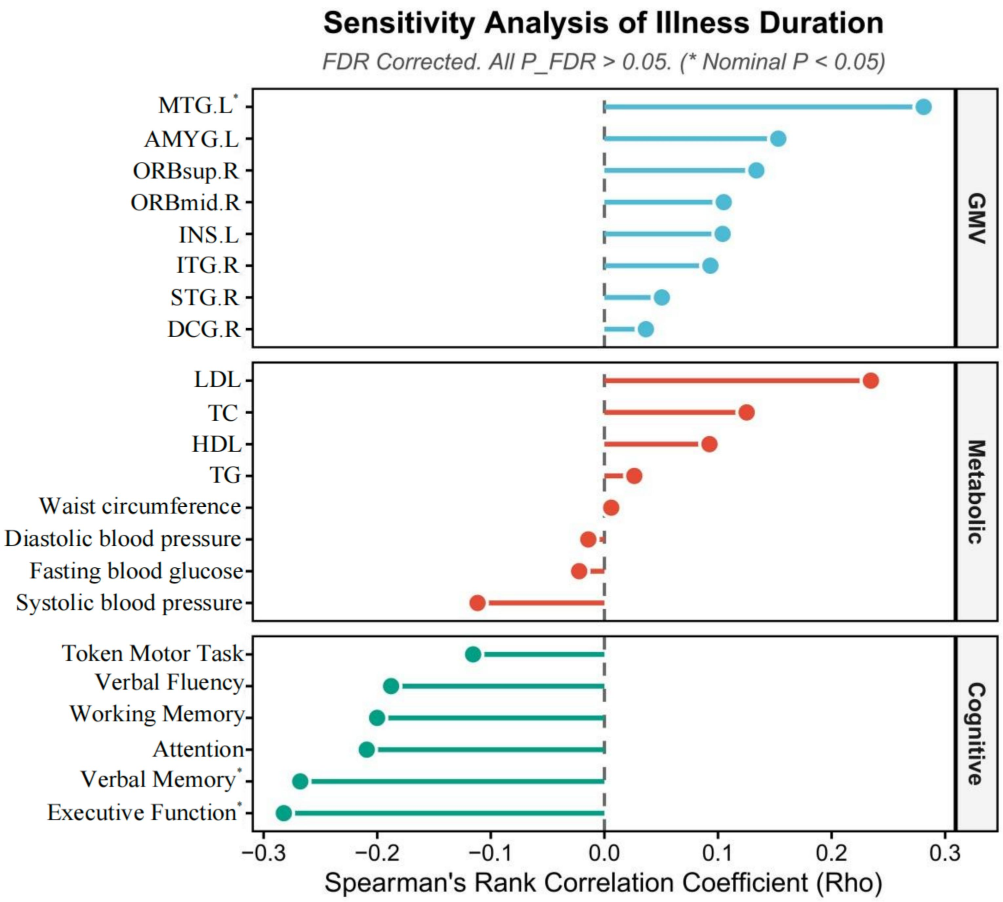

Note 1:R/L-right/left hemisphere; MTG-Middle temporal gyrus; STG-Superior temporal gyrus; AMYG-Amygdala; ORBsup-Superior frontal gyrus, orbital part; ITG-Inferior temporal gyrus; INS-Insula; ORBmid-Middle frontal gyrus, orbital part; DCG-Median cingulate and paracingulate gyri; LDL-Low density lipoprotein; TC-Total cholesterol ; HDL-High-density lipoprotein ; TG-Triglycerides; GMV-gray matter volume.

Table 1. Interaction Analysis (Age × Group, Sex × Group) for GMV and Cognition

Variables	Interaction Effect	F-value	Raw p-value	FDR-corrected p-value
INS.L	Group x Age	8.930	0.004	0.120
	Group x Gender	0.038	0.846	0.947
AMYG.L	Group x Age	1.402	0.242	0.873
	Group x Gender	0.319	0.574	0.873
DCG.R	Group x Age	1.587	0.213	0.873
	Group x Gender	0.140	0.710	0.904
ORBsup.R	Group x Age	0.495	0.485	0.873
	Group x Gender	0.296	0.589	0.873
ORBmid.R	Group x Age	0.001	0.971	0.971
	Group x Gender	3.294	0.075	0.873
MTG.L	Group x Age	0.046	0.831	0.947
	Group x Gender	0.011	0.915	0.949
ITG.R	Group x Age	0.290	0.592	0.873
	Group x Gender	2.026	0.161	0.873
STG.R	Group x Age	0.797	0.376	0.873
	Group x Gender	2.667	0.109	0.873
Verbal Memory	Group x Age	0.367	0.547	0.873
	Group x Gender	0.144	0.706	0.904
Working Memory	Group x Age	1.274	0.264	0.873
	Group x Gender	1.494	0.227	0.873
Token Motor Task	Group x Age	0.405	0.527	0.873
	Group x Gender	0.175	0.677	0.904
Verbal Fluency	Group x Age	0.080	0.778	0.947
	Group x Gender	0.733	0.396	0.873
Attention	Group x Age	0.369	0.546	0.873
	Group x Gender	0.311	0.579	0.873
Executive Function	Group x Age	0.683	0.412	0.873
	Group x Gender	0.020	0.888	0.949

Note 2: R/L-right/left hemisphere; MTG-Middle temporal gyrus; STG-Superior temporal gyrus; AMYG-Amygdala; ORBsup-Superior frontal gyrus, orbital part; ITG-Inferior temporal gyrus; INS-Insula; ORBmid-Middle frontal gyrus, orbital part; DCG-Median cingulate and paracingulate gyri.
